# Spontaneous pneumomediastinum and surgical emphysema presenting as rhinolalia following ecstasy ingestion: a case report

**DOI:** 10.1186/s13256-024-04618-9

**Published:** 2024-06-28

**Authors:** Caterina Prada, Max Ross

**Affiliations:** Whiston Hospital, Mersey and West Lancashire Teaching Hospitals NHS Trust, Rainhill, Prescot UK

**Keywords:** Case report, Rhinolalia, Spontaneous pneumomediastinum, Surgical emphysema, Ecstasy

## Abstract

**Background:**

We present a unique case of rhinolalia as the first recognizable sign of spontaneous pneumomediastinum and surgical emphysema following drug use.

**Case presentation:**

This case presents a 17-year-old white male experiencing rhinolalia following ecstasy ingestion at a rave. Subsequent chest X-ray revealed extensive surgical emphysema, along with a continuous diaphragm sign indicative of pneumomediastinum. Computed tomography confirmed the diagnosis. The patient was managed conservatively with strict monitoring and 6 hourly electrocardiograms. Follow-up computed tomography on day 3 showed resolution of pneumomediastinum and surgical emphysema, and the patient was safely discharged. Notably, the patient experienced a temporary rhinolalia during the acute phase, which resolved spontaneously as his condition improved.

**Conclusions:**

This case underscores the importance of considering spontaneous pneumomediastinum and surgical emphysema in the differential diagnosis of young individuals presenting with acute symptoms after drug use.

## Background

3,4-methylenedioxymethamphetamine (MDMA), also known as ecstasy, is an illicit drug widely used for recreational purposes. Originally developed by a German pharmaceutical company in 1912, it has gained popularity due to its mood-enhancing effects and ability to induce a sense of euphoria [1]. However, the use of ecstasy carries numerous risks, including cardiovascular complications, hyperthermia, dehydration, and serotonin syndrome. Additionally, rare respiratory complications such as pneumothorax, surgical emphysema, and pneumomediastinum have been documented in the medical literature [2–4]. These complications often manifest as chest pain, dyspnea, or cough. In this case report, we present the unique case of a 17-year-old male who developed spontaneous pneumomediastinum and surgical emphysema following ecstasy ingestion, with an initial presentation characterized by rhinolalia.

## Case presentation

### Patient information

A 17-year-old white male presented to the emergency department (ED) of a district general hospital after experiencing rhinolalia following ingestion of ecstasy at a rave. The patient later complained of left-sided pleuritic chest pain and dyspnea. He had no past medical history and denied smoking.

### Clinical findings and diagnostic assessment

On examination, there were no signs of airway compromise, but bilateral wheezing was heard on auscultation. Tactile crepitus was observed over the subclavian region and superior thorax. Notably, the patient’s voice had changed, becoming high-pitched with a significant hyponasal quality and poor resonance.

**Fig. 1:**
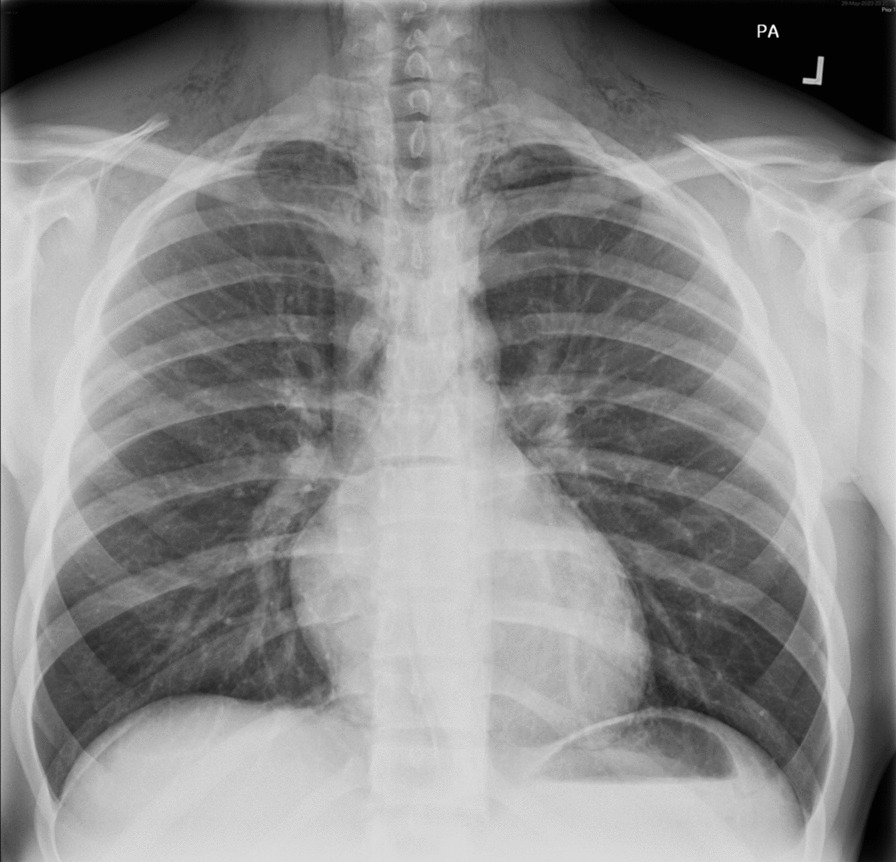
Posteroanterior chest radiograph demonstrating widespread surgical emphysema over the superior thorax and neck bilaterally

**Fig. 2:**
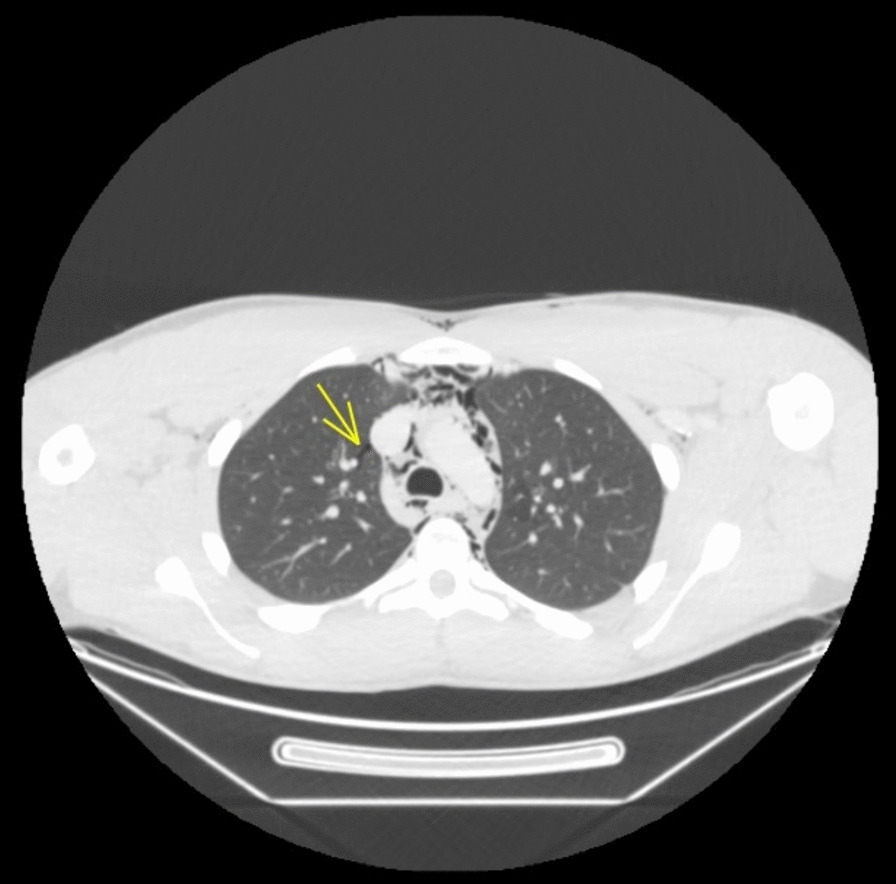
Computed tomography axial image of chest demonstrating pneumomediastinum and mild pneumopericardium. Yellow arrow: pneumomediastinum within the left superior mediastinum

Blood tests were unremarkable. A chest X-ray revealed extensive surgical emphysema over the superior thorax and neck bilaterally (Fig. 1), along with a continuous diaphragm sign indicative of pneumomediastinum. Subsequent computed tomography (CT) of the neck with oral contrast confirmed widespread presence of surgical emphysema, pneumomediastinum, and mild pneumopericardium (Fig. 2). No cause was identified. A water-soluble contrast swallow test ruled out Boerhaave syndrome.

### Therapeutic intervention and outcomes

The cardiothoracic surgeons recommended admitting the patient for close monitoring with 6-hourly electrocardiograms (ECG), opting for conservative management. On the third day of admission, a follow-up CT of th neck with oral contrast revealed improvement of the pneumomediastinum and subcutaneous emphysema. Considering this positive outcome and the stability of the patient, he was discharged the same day.

## Discussion

Spontaneous pneumomediastinum (SPM) is defined as free air in the mediastinum, unrelated to trauma [4], primarily affecting young men. It is estimated to have a prevalence of 1 in 30,000 ED admissions [5] and was first described by Hamman in 1939 [6]. The most reported symptoms of SPM include chest pain, dyspnea, and subcutaneous emphysema [7]. In rare cases, SPM can present with unusual symptoms of rhinolalia, dysphagia, and pharyngitis [8].

Ecstasy, a potent serotonergic agent, stimulates the release of serotonin, dopamine, and noradrenaline. In turn, this leads to a sympathetic response causing increased heart rate, blood pressure, and bronchodilation [1]. While these physiological changes may increase the risk of SPM, the pathophysiology of SPM in relation to ecstasy use is believed to be associated with the drug’s impact on stamina [9]. Ecstasy is commonly consumed at parties where activities such as shouting, singing, and strenuous activities are performed, resulting in increased Valsalva maneuvres. This, therefore, raises intrathoracic pressure, leading to marginal alveolar rupture and free air dissecting to the interstitial space toward the mediastinum [9].

In our case study, the initial clinical manifestation was rhinolalia. Typically, rhinolalia indicates anatomical or functional abnormalities of the nasopharynx. However, in rare instances, rhinolalia can suggest dissection of air from the mediastinum to the subcutaneous tissues of the head and neck [10]. While there have been some documented association between SPM and rhinolalia [11], it is often an underappreciated sign for cardiothoracic surgeons [12]. Consequently, some cases of SPM are inevitably missed, potentially leading to life-threatening complications such as pneumothorax, pneumopericardium, cardiac tamponade, and mediastinitis [13]. This highlights the importance of recognizing all clinical signs of SPM.

## Conclusion

Our case highlights the importance of recognizing rhinolalia as a clinical sign of SPM, which holds relevance across various medical and surgical specialties. It is crucial to include SPM in the differential diagnosis when encountering individuals with new-onset, abrupt rhinolalia.

Furthermore, we have presented another case of a young individual who developed SPM and surgical emphysema following ecstasy use. This contributes valuable information to a limited body of evidence concerning the prevalence and management of SPM in the context of ecstasy ingestion. Additionally, we have highlighted rhinolalia as a rare presentation of SPM.

## Learning points

1. Maintain a high index of suspicion for SPM and subcutaneous emphysema in patients who report the use of ecstasy and/or cocaine.
2. When there is clinical suspicion of SPM, comprehensive evaluation including imaging modalities such as CT scans of the head, neck, and thorax, along with appropriate laboratory tests, should be pursued. Fluoroscopy can aid in ruling out Boerhaave syndrome.
3. Consider the potential presence of SPM or nasopharyngeal injury in patients presenting with new sudden onset rhinolalia.

## Data Availability

Anonymous participant data are available upon reasonable request from Dr. Caterina Prada and Dr. Max Ross. Reuse is not permitted unless permission is explicitly granted by the authors.

## Abbreviations

MDMA: 3,4-Methylenedioxymethamphetamine
ED: Emergency department
CT: Computed tomography
ECG: Electrocardiogram
SPM: Spontaneous pneumomediastinum
